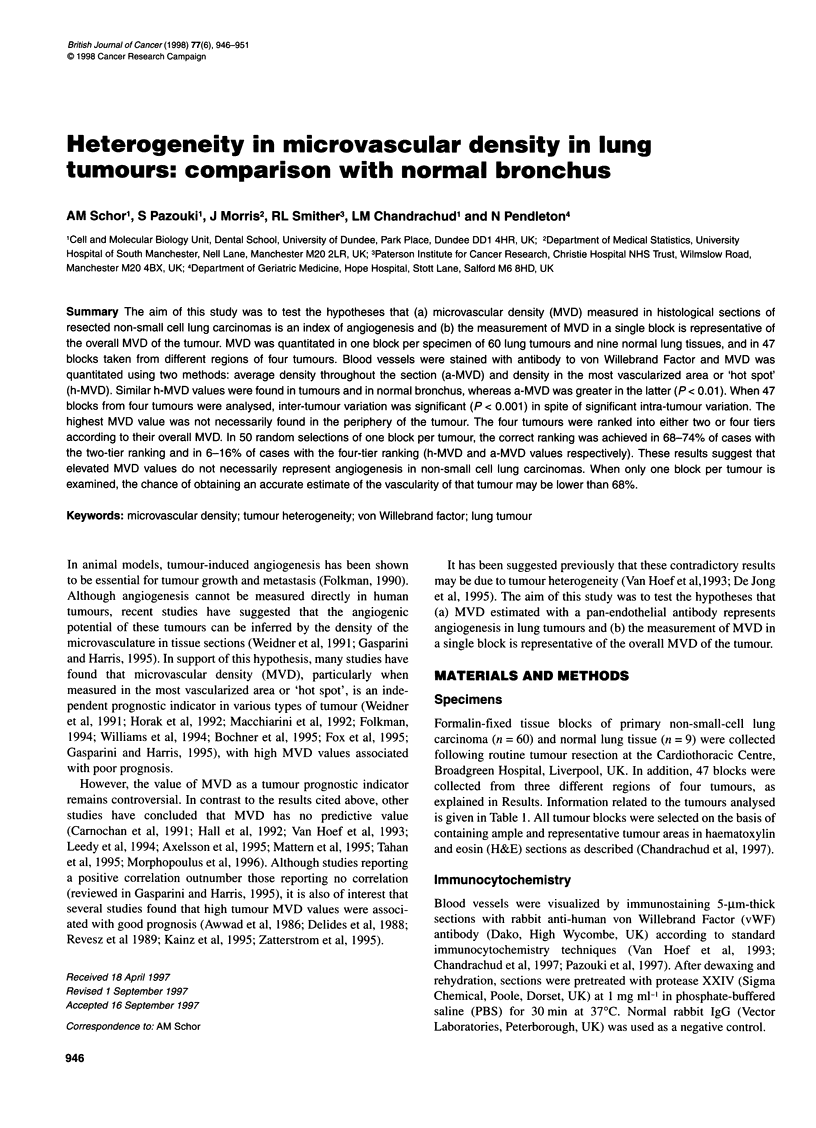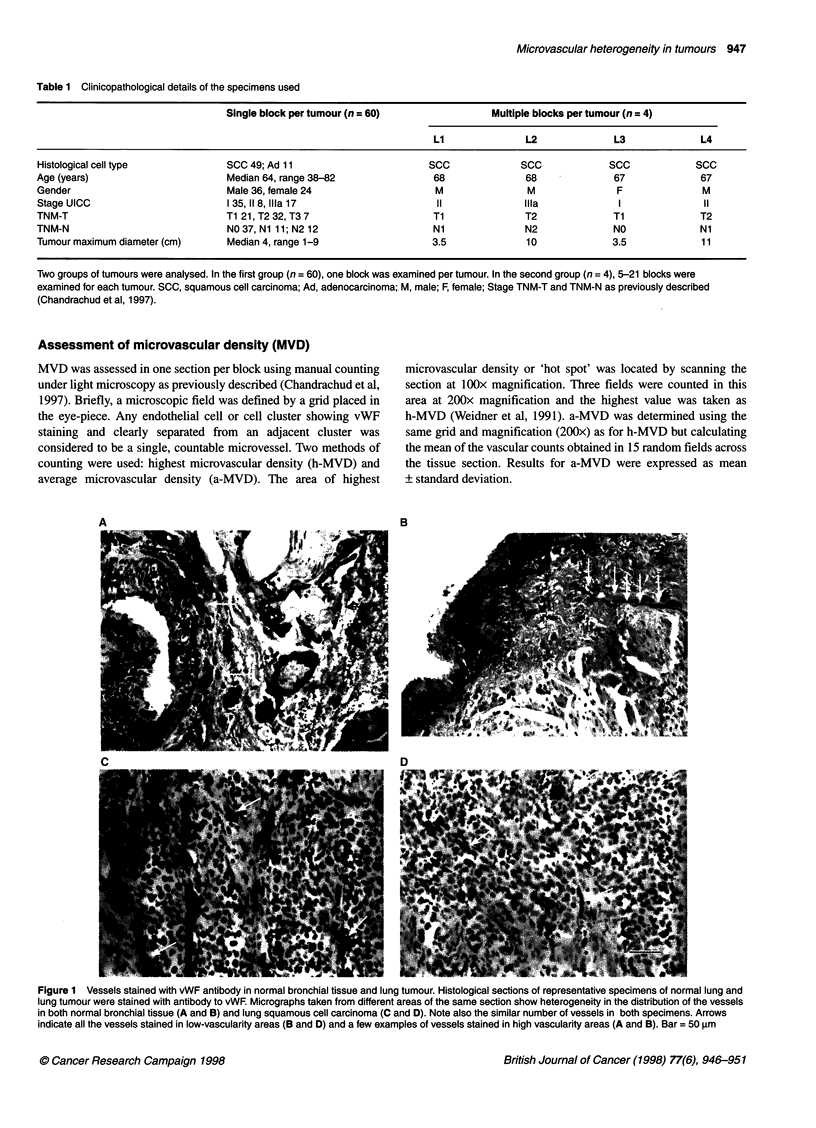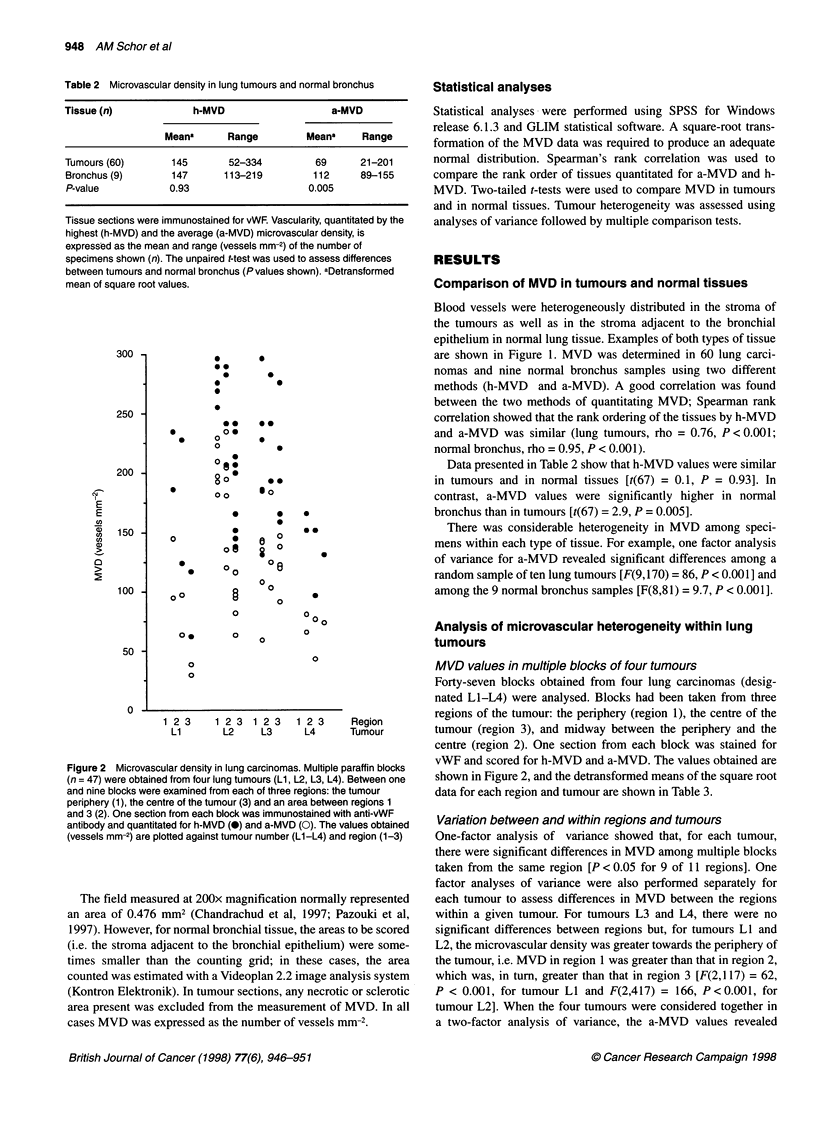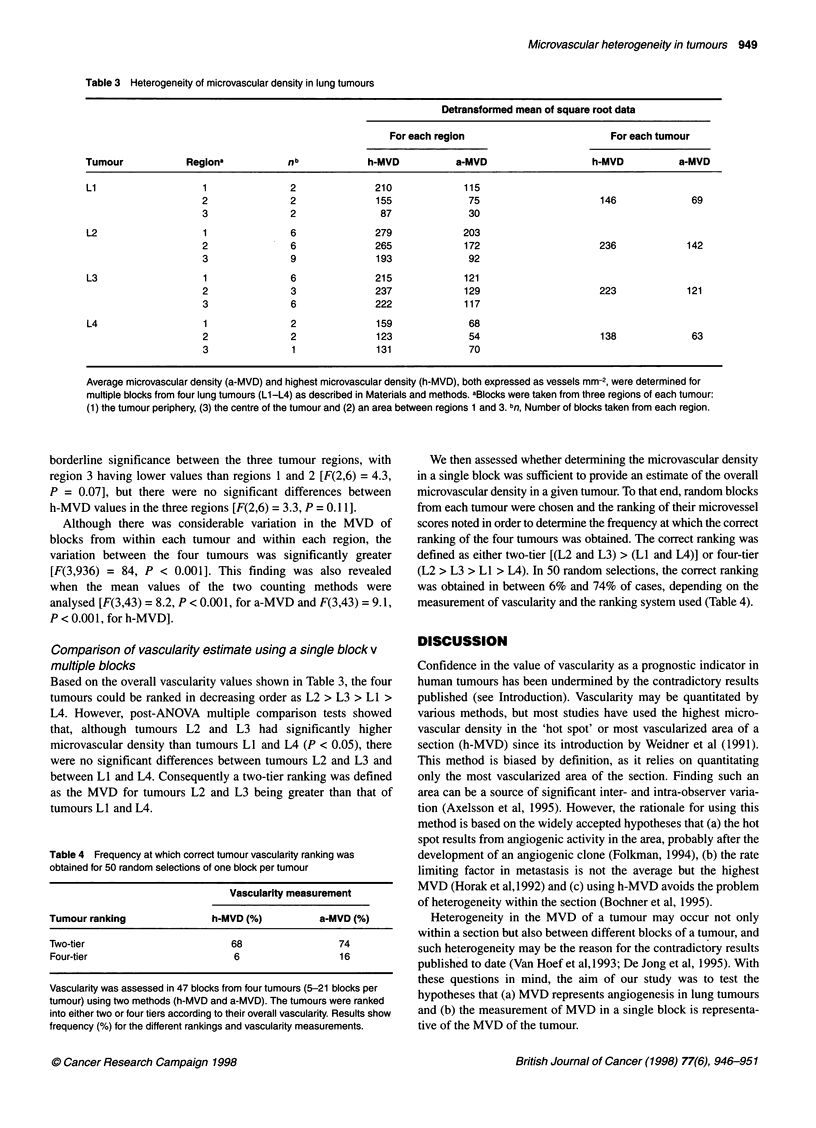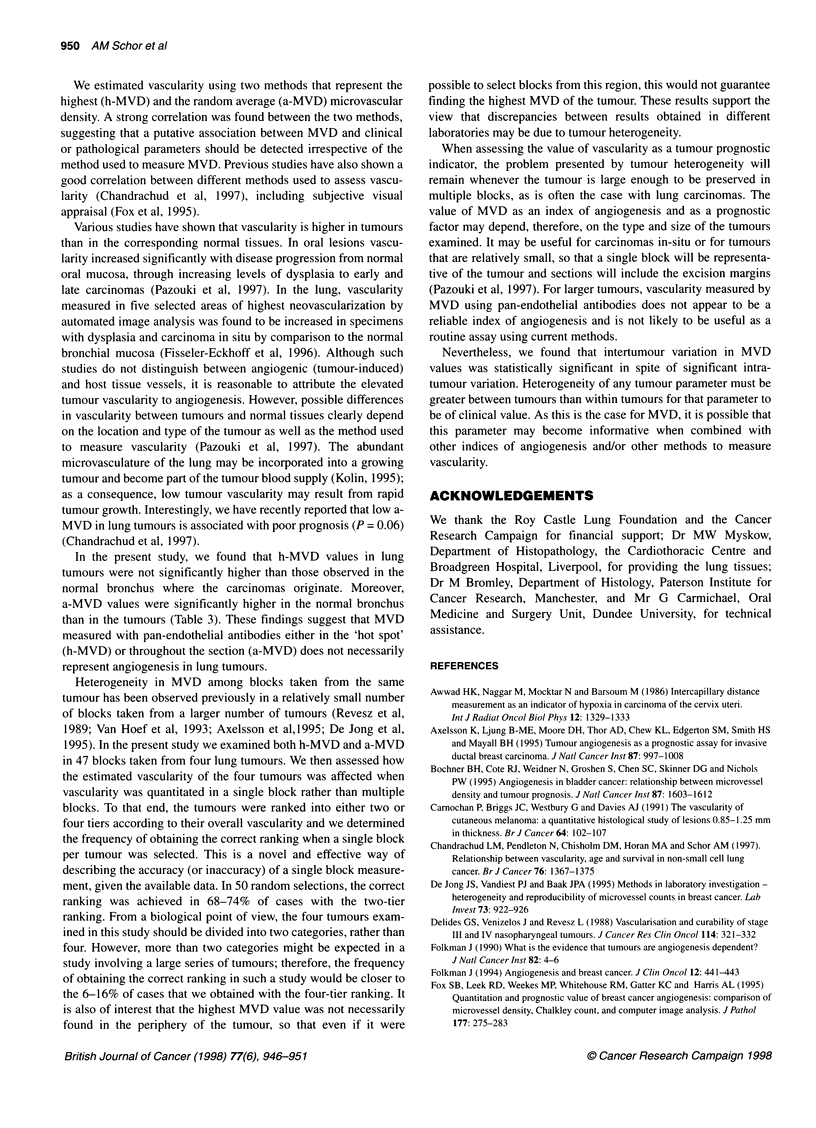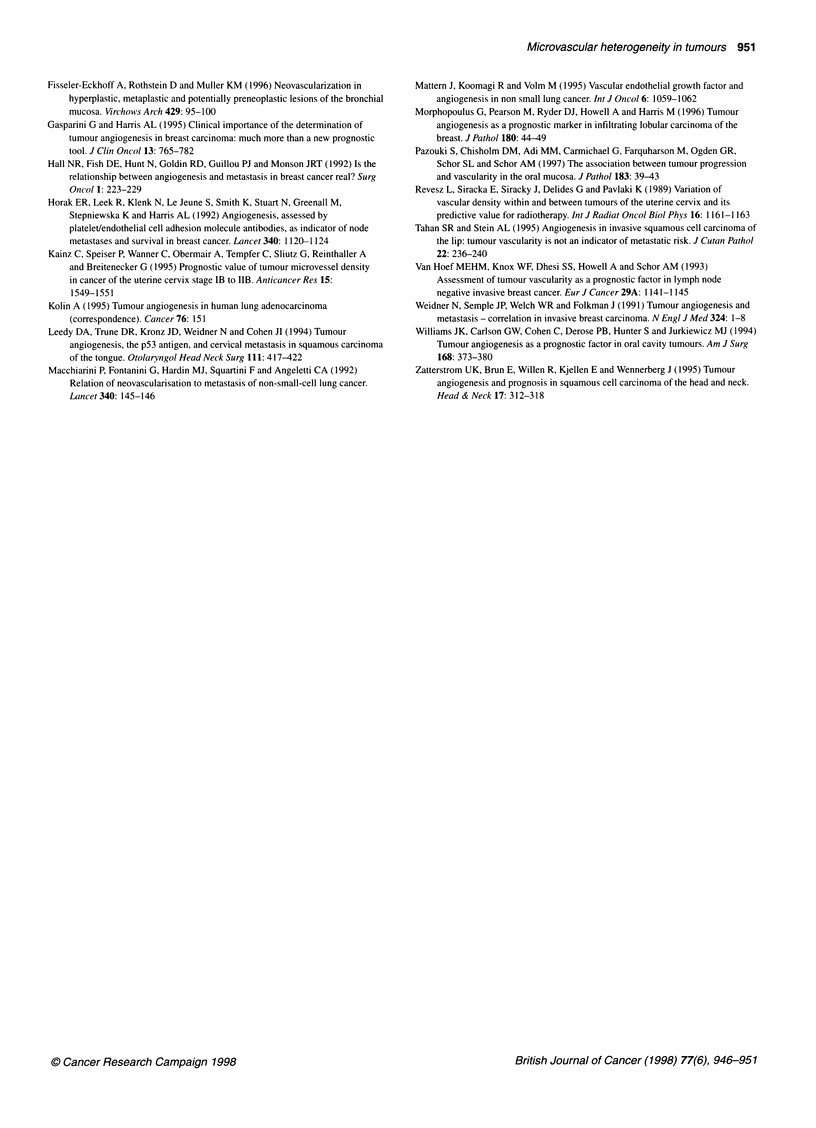# Heterogeneity in microvascular density in lung tumours: comparison with normal bronchus.

**DOI:** 10.1038/bjc.1998.156

**Published:** 1998-03

**Authors:** A. M. Schor, S. Pazouki, J. Morris, R. L. Smither, L. M. Chandrachud, N. Pendleton

**Affiliations:** Cell and Molecular Biology Unit, Dental School, University of Dundee, UK.

## Abstract

**Images:**


British Journal of Cancer (1998) 77(6), 946–951
© 1998 Cancer Research Campaign

### Heterogeneity in microvascular density in lung tumours: comparison with normal bronchus

**AM Schor[1], S Pazouki[1], J Morris[2], RL Smither[3], LM Chandrachud[1] and N Pendleton[4]**

[1]Cell and Molecular Biology Unit, Dental School, University of Dundee, Park Place, Dundee DD1 4HR, UK; [2]Department of Medical Statistics, University Hospital of South Manchester, Nell Lane, Manchester M20 2LR, UK; [3]Paterson Institute for Cancer Research, Christie Hospital NHS Trust, Wilmslow Road, Manchester M20 4BX, UK; [4]Department of Geriatric Medicine, Hope Hospital, Stott Lane, Salford M6 8HD, UK

**Summary** The aim of this study was to test the hypotheses that (a) microvascular density (MVD) measured in histological sections of resected non-small cell lung carcinomas is an index of angiogenesis and (b) the measurement of MVD in a single block is representative of the overall MVD of the tumour. MVD was quantitated in one block per specimen of 60 lung tumours and nine normal lung tissues, and in 47 blocks taken from different regions of four tumours. Blood vessels were stained with antibody to von Willebrand Factor and MVD was quantitated using two methods: average density throughout the section (a-MVD) and density in the most vascularized area or 'hot spot' (h-MVD). Similar h-MVD values were found in tumours and in normal bronchus, whereas a-MVD was greater in the latter ($P < 0.01$). When 47 blocks from four tumours were analysed, inter-tumour variation was significant ($P < 0.001$) in spite of significant intra-tumour variation. The highest MVD value was not necessarily found in the periphery of the tumour. The four tumours were ranked into either two or four tiers according to their overall MVD. In 50 random selections of one block per tumour, the correct ranking was achieved in 68–74% of cases with the two-tier ranking and in 6–16% of cases with the four-tier ranking (h-MVD and a-MVD values respectively). These results suggest that elevated MVD values do not necessarily represent angiogenesis in non-small cell lung carcinomas. When only one block per tumour is examined, the chance of obtaining an accurate estimate of the vascularity of that tumour may be lower than 68%.

**Keywords:** microvascular density; tumour heterogeneity; von Willebrand factor; lung tumour

In animal models, tumour-induced angiogenesis has been shown to be essential for tumour growth and metastasis (Folkman, 1990). Although angiogenesis cannot be measured directly in human tumours, recent studies have suggested that the angiogenic potential of these tumours can be inferred by the density of the microvasculature in tissue sections (Weidner et al, 1991; Gasparini and Harris, 1995). In support of this hypothesis, many studies have found that microvascular density (MVD), particularly when measured in the most vascularized area or 'hot spot', is an independent prognostic indicator in various types of tumour (Weidner et al, 1991; Horak et al, 1992; Macchiarini et al, 1992; Folkman, 1994; Williams et al, 1994; Bochner et al, 1995; Fox et al, 1995; Gasparini and Harris, 1995), with high MVD values associated with poor prognosis.

However, the value of MVD as a tumour prognostic indicator remains controversial. In contrast to the results cited above, other studies have concluded that MVD has no predictive value (Carnochan et al, 1991; Hall et al, 1992; Van Hoef et al, 1993; Leedy et al, 1994; Axelsson et al, 1995; Mattern et al, 1995; Tahan et al, 1995; Morphopoulus et al, 1996). Although studies reporting a positive correlation outnumber those reporting no correlation (reviewed in Gasparini and Harris, 1995), it is also of interest that several studies found that high tumour MVD values were associated with good prognosis (Awwad et al, 1986; Delides et al, 1988; Revesz et al 1989; Kainz et al, 1995; Zatterstrom et al, 1995).

*Received 18 April 1997*
*Revised 1 September 1997*
*Accepted 16 September 1997*

*Correspondence to:* AM Schor

It has been suggested previously that these contradictory results may be due to tumour heterogeneity (Van Hoef et al,1993; De Jong et al, 1995). The aim of this study was to test the hypotheses that (a) MVD estimated with a pan-endothelial antibody represents angiogenesis in lung tumours and (b) the measurement of MVD in a single block is representative of the overall MVD of the tumour.

### MATERIALS AND METHODS

### Specimens

Formalin-fixed tissue blocks of primary non-small-cell lung carcinoma ($n = 60$) and normal lung tissue ($n = 9$) were collected following routine tumour resection at the Cardiothoracic Centre, Broadgreen Hospital, Liverpool, UK. In addition, 47 blocks were collected from three different regions of four tumours, as explained in Results. Information related to the tumours analysed is given in Table 1. All tumour blocks were selected on the basis of containing ample and representative tumour areas in haematoxylin and eosin (H&E) sections as described (Chandrachud et al, 1997).

### Immunocytochemistry

Blood vessels were visualized by immunostaining 5-μm-thick sections with rabbit anti-human von Willebrand Factor (vWF) antibody (Dako, High Wycombe, UK) according to standard immunocytochemistry techniques (Van Hoef et al, 1993; Chandrachud et al, 1997; Pazouki et al, 1997). After dewaxing and rehydration, sections were pretreated with protease XXIV (Sigma Chemical, Poole, Dorset, UK) at 1 mg $ml^{-1}$ in phosphate-buffered saline (PBS) for 30 min at 37°C. Normal rabbit IgG (Vector Laboratories, Peterborough, UK) was used as a negative control.

**Table 1** Clinicopathological details of the specimens used

| | Single block per tumour (*n* = 60) | Multiple blocks per tumour (*n* = 4) | | | |
|---|---|---|---|---|---|
| | | L1 | L2 | L3 | L4 |
| Histological cell type | SCC 49; Ad 11 | SCC | SCC | SCC | SCC |
| Age (years) | Median 64, range 38–82 | 68 | 68 | 67 | 67 |
| Gender | Male 36, female 24 | M | M | F | M |
| Stage UICC | I 35, II 8, IIIa 17 | II | IIIa | I | II |
| TNM-T | T1 21, T2 32, T3 7 | T1 | T2 | T1 | T2 |
| TNM-N | N0 37, N1 11; N2 12 | N1 | N2 | N0 | N1 |
| Tumour maximum diameter (cm) | Median 4, range 1–9 | 3.5 | 10 | 3.5 | 11 |

Two groups of tumours were analysed. In the first group (*n* = 60), one block was examined per tumour. In the second group (*n* = 4), 5–21 blocks were examined for each tumour. SCC, squamous cell carcinoma; Ad, adenocarcinoma; M, male; F, female; Stage TNM-T and TNM-N as previously described (Chandrachud et al, 1997).

### Assessment of microvascular density (MVD)

MVD was assessed in one section per block using manual counting under light microscopy as previously described (Chandrachud et al, 1997). Briefly, a microscopic field was defined by a grid placed in the eye-piece. Any endothelial cell or cell cluster showing vWF staining and clearly separated from an adjacent cluster was considered to be a single, countable microvessel. Two methods of counting were used: highest microvascular density (h-MVD) and average microvascular density (a-MVD). The area of highest microvascular density or 'hot spot' was located by scanning the section at 100× magnification. Three fields were counted in this area at 200× magnification and the highest value was taken as h-MVD (Weidner et al, 1991). a-MVD was determined using the same grid and magnification (200×) as for h-MVD but calculating the mean of the vascular counts obtained in 15 random fields across the tissue section. Results for a-MVD were expressed as mean ± standard deviation.

**Figure 1** Vessels stained with vWF antibody in normal bronchial tissue and lung tumour. Histological sections of representative specimens of normal lung and lung tumour were stained with antibody to vWF. Micrographs taken from different areas of the same section show heterogeneity in the distribution of the vessels in both normal bronchial tissue (**A** and **B**) and lung squamous cell carcinoma (**C** and **D**). Note also the similar number of vessels in both specimens. Arrows indicate all the vessels stained in low-vascularity areas (**B** and **D**) and a few examples of vessels stained in high vascularity areas (**A** and **B**). Bar = 50 µm

**Table 2** Microvascular density in lung tumours and normal bronchus

| Tissue (*n*) | h-MVD | | a-MVD | |
|---|---|---|---|---|
| | Mean[a] | Range | Mean[a] | Range |
| Tumours (60) | 145 | 52–334 | 69 | 21–201 |
| Bronchus (9) | 147 | 113–219 | 112 | 89–155 |
| *P*-value | 0.93 | | 0.005 | |

Tissue sections were immunostained for vWF. Vascularity, quantitated by the highest (h-MVD) and the average (a-MVD) microvascular density, is expressed as the mean and range (vessels mm$^{-2}$) of the number of specimens shown (*n*). The unpaired *t*-test was used to assess differences between tumours and normal bronchus (*P* values shown). [a]Detransformed mean of square root values.

**Figure 2** Microvascular density in lung carcinomas. Multiple paraffin blocks (*n* = 47) were obtained from four lung tumours (L1, L2, L3, L4). Between one and nine blocks were examined from each of three regions: the tumour periphery (1), the centre of the tumour (3) and an area between regions 1 and 3 (2). One section from each block was immunostained with anti-vWF antibody and quantitated for h-MVD (●) and a-MVD (○). The values obtained (vessels mm$^{-2}$) are plotted against tumour number (L1–L4) and region (1–3)

The field measured at 200× magnification normally represented an area of 0.476 mm$^2$ (Chandrachud et al, 1997; Pazouki et al, 1997). However, for normal bronchial tissue, the areas to be scored (i.e. the stroma adjacent to the bronchial epithelium) were sometimes smaller than the counting grid; in these cases, the area counted was estimated with a Videoplan 2.2 image analysis system (Kontron Elektronik). In tumour sections, any necrotic or sclerotic area present was excluded from the measurement of MVD. In all cases MVD was expressed as the number of vessels mm$^{-2}$.

### Statistical analyses

Statistical analyses were performed using SPSS for Windows release 6.1.3 and GLIM statistical software. A square-root transformation of the MVD data was required to produce an adequate normal distribution. Spearman's rank correlation was used to compare the rank order of tissues quantitated for a-MVD and h-MVD. Two-tailed *t*-tests were used to compare MVD in tumours and in normal tissues. Tumour heterogeneity was assessed using analyses of variance followed by multiple comparison tests.

### RESULTS

### Comparison of MVD in tumours and normal tissues

Blood vessels were heterogeneously distributed in the stroma of the tumours as well as in the stroma adjacent to the bronchial epithelium in normal lung tissue. Examples of both types of tissue are shown in Figure 1. MVD was determined in 60 lung carcinomas and nine normal bronchus samples using two different methods (h-MVD and a-MVD). A good correlation was found between the two methods of quantitating MVD; Spearman rank correlation showed that the rank ordering of the tissues by h-MVD and a-MVD was similar (lung tumours, rho = 0.76, $P < 0.001$; normal bronchus, rho = 0.95, $P < 0.001$).

Data presented in Table 2 show that h-MVD values were similar in tumours and in normal tissues [$t(67) = 0.1$, $P = 0.93$]. In contrast, a-MVD values were significantly higher in normal bronchus than in tumours [$t(67) = 2.9$, $P = 0.005$].

There was considerable heterogeneity in MVD among specimens within each type of tissue. For example, one factor analysis of variance for a-MVD revealed significant differences among a random sample of ten lung tumours [$F(9,170) = 86$, $P < 0.001$] and among the 9 normal bronchus samples [$F(8,81) = 9.7$, $P < 0.001$].

### Analysis of microvascular heterogeneity within lung tumours

### *MVD values in multiple blocks of four tumours*

Forty-seven blocks obtained from four lung carcinomas (designated L1–L4) were analysed. Blocks had been taken from three regions of the tumour: the periphery (region 1), the centre of the tumour (region 3), and midway between the periphery and the centre (region 2). One section from each block was stained for vWF and scored for h-MVD and a-MVD. The values obtained are shown in Figure 2, and the detransformed means of the square root data for each region and tumour are shown in Table 3.

### *Variation between and within regions and tumours*

One-factor analysis of variance showed that, for each tumour, there were significant differences in MVD among multiple blocks taken from the same region [$P < 0.05$ for 9 of 11 regions]. One factor analyses of variance were also performed separately for each tumour to assess differences in MVD between the regions within a given tumour. For tumours L3 and L4, there were no significant differences between regions but, for tumours L1 and L2, the microvascular density was greater towards the periphery of the tumour, i.e. MVD in region 1 was greater than that in region 2, which was, in turn, greater than that in region 3 [$F(2,117) = 62$, $P < 0.001$, for tumour L1 and $F(2,417) = 166$, $P < 0.001$, for tumour L2]. When the four tumours were considered together in a two-factor analysis of variance, the a-MVD values revealed

 © Cancer Research Campaign 1998

**Table 3** Heterogeneity of microvascular density in lung tumours

| | | | Detransformed mean of square root data | | | |
|---|---|---|---|---|---|---|
| | | | For each region | | For each tumour | |
| Tumour | Region[a] | $n^b$ | h-MVD | a-MVD | h-MVD | a-MVD |
| L1 | 1 | 2 | 210 | 115 | | |
| | 2 | 2 | 155 | 75 | 146 | 69 |
| | 3 | 2 | 87 | 30 | | |
| L2 | 1 | 6 | 279 | 203 | | |
| | 2 | 6 | 265 | 172 | 236 | 142 |
| | 3 | 9 | 193 | 92 | | |
| L3 | 1 | 6 | 215 | 121 | | |
| | 2 | 3 | 237 | 129 | 223 | 121 |
| | 3 | 6 | 222 | 117 | | |
| L4 | 1 | 2 | 159 | 68 | | |
| | 2 | 2 | 123 | 54 | 138 | 63 |
| | 3 | 1 | 131 | 70 | | |

Average microvascular density (a-MVD) and highest microvascular density (h-MVD), both expressed as vessels $mm^{-2}$, were determined for multiple blocks from four lung tumours (L1–L4) as described in Materials and methods. [a]Blocks were taken from three regions of each tumour: (1) the tumour periphery, (3) the centre of the tumour and (2) an area between regions 1 and 3. [b]*n*, Number of blocks taken from each region.

borderline significance between the three tumour regions, with region 3 having lower values than regions 1 and 2 [$F(2,6) = 4.3$, $P = 0.07$], but there were no significant differences between h-MVD values in the three regions [$F(2,6) = 3.3$, $P = 0.11$].

Although there was considerable variation in the MVD of blocks from within each tumour and within each region, the variation between the four tumours was significantly greater [$F(3,936) = 84$, $P < 0.001$]. This finding was also revealed when the mean values of the two counting methods were analysed [$F(3,43) = 8.2$, $P < 0.001$, for a-MVD and $F(3,43) = 9.1$, $P < 0.001$, for h-MVD].

### *Comparison of vascularity estimate using a single block* v *multiple blocks*

Based on the overall vascularity values shown in Table 3, the four tumours could be ranked in decreasing order as L2 > L3 > L1 > L4. However, post-ANOVA multiple comparison tests showed that, although tumours L2 and L3 had significantly higher microvascular density than tumours L1 and L4 ($P < 0.05$), there were no significant differences between tumours L2 and L3 and between L1 and L4. Consequently a two-tier ranking was defined as the MVD for tumours L2 and L3 being greater than that of tumours L1 and L4.

**Table 4** Frequency at which correct tumour vascularity ranking was obtained for 50 random selections of one block per tumour

| | Vascularity measurement | |
|---|---|---|
| Tumour ranking | h-MVD (%) | a-MVD (%) |
| Two-tier | 68 | 74 |
| Four-tier | 6 | 16 |

Vascularity was assessed in 47 blocks from four tumours (5–21 blocks per tumour) using two methods (h-MVD and a-MVD). The tumours were ranked into either two or four tiers according to their overall vascularity. Results show frequency (%) for the different rankings and vascularity measurements.

We then assessed whether determining the microvascular density in a single block was sufficient to provide an estimate of the overall microvascular density in a given tumour. To that end, random blocks from each tumour were chosen and the ranking of their microvessel scores noted in order to determine the frequency at which the correct ranking of the four tumours was obtained. The correct ranking was defined as either two-tier [(L2 and L3) > (L1 and L4)] or four-tier (L2 > L3 > L1 > L4). In 50 random selections, the correct ranking was obtained in between 6% and 74% of cases, depending on the measurement of vascularity and the ranking system used (Table 4).

### DISCUSSION

Confidence in the value of vascularity as a prognostic indicator in human tumours has been undermined by the contradictory results published (see Introduction). Vascularity may be quantitated by various methods, but most studies have used the highest microvascular density in the 'hot spot' or most vascularized area of a section (h-MVD) since its introduction by Weidner et al (1991). This method is biased by definition, as it relies on quantitating only the most vascularized area of the section. Finding such an area can be a source of significant inter- and intra-observer variation (Axelsson et al, 1995). However, the rationale for using this method is based on the widely accepted hypotheses that (a) the hot spot results from angiogenic activity in the area, probably after the development of an angiogenic clone (Folkman, 1994), (b) the rate limiting factor in metastasis is not the average but the highest MVD (Horak et al,1992) and (c) using h-MVD avoids the problem of heterogeneity within the section (Bochner et al, 1995).

Heterogeneity in the MVD of a tumour may occur not only within a section but also between different blocks of a tumour, and such heterogeneity may be the reason for the contradictory results published to date (Van Hoef et al,1993; De Jong et al, 1995). With these questions in mind, the aim of our study was to test the hypotheses that (a) MVD represents angiogenesis in lung tumours and (b) the measurement of MVD in a single block is representative of the MVD of the tumour.

© Cancer Research Campaign 1998

We estimated vascularity using two methods that represent the highest (h-MVD) and the random average (a-MVD) microvascular density. A strong correlation was found between the two methods, suggesting that a putative association between MVD and clinical or pathological parameters should be detected irrespective of the method used to measure MVD. Previous studies have also shown a good correlation between different methods used to assess vascularity (Chandrachud et al, 1997), including subjective visual appraisal (Fox et al, 1995).

Various studies have shown that vascularity is higher in tumours than in the corresponding normal tissues. In oral lesions vascularity increased significantly with disease progression from normal oral mucosa, through increasing levels of dysplasia to early and late carcinomas (Pazouki et al, 1997). In the lung, vascularity measured in five selected areas of highest neovascularization by automated image analysis was found to be increased in specimens with dysplasia and carcinoma in situ by comparison to the normal bronchial mucosa (Fisseler-Eckhoff et al, 1996). Although such studies do not distinguish between angiogenic (tumour-induced) and host tissue vessels, it is reasonable to attribute the elevated tumour vascularity to angiogenesis. However, possible differences in vascularity between tumours and normal tissues clearly depend on the location and type of the tumour as well as the method used to measure vascularity (Pazouki et al, 1997). The abundant microvasculature of the lung may be incorporated into a growing tumour and become part of the tumour blood supply (Kolin, 1995); as a consequence, low tumour vascularity may result from rapid tumour growth. Interestingly, we have recently reported that low a-MVD in lung tumours is associated with poor prognosis ($P = 0.06$) (Chandrachud et al, 1997).

In the present study, we found that h-MVD values in lung tumours were not significantly higher than those observed in the normal bronchus where the carcinomas originate. Moreover, a-MVD values were significantly higher in the normal bronchus than in the tumours (Table 3). These findings suggest that MVD measured with pan-endothelial antibodies either in the 'hot spot' (h-MVD) or throughout the section (a-MVD) does not necessarily represent angiogenesis in lung tumours.

Heterogeneity in MVD among blocks taken from the same tumour has been observed previously in a relatively small number of blocks taken from a larger number of tumours (Revesz et al, 1989; Van Hoef et al, 1993; Axelsson et al,1995; De Jong et al, 1995). In the present study we examined both h-MVD and a-MVD in 47 blocks taken from four lung tumours. We then assessed how the estimated vascularity of the four tumours was affected when vascularity was quantitated in a single block rather than multiple blocks. To that end, the tumours were ranked into either two or four tiers according to their overall vascularity and we determined the frequency of obtaining the correct ranking when a single block per tumour was selected. This is a novel and effective way of describing the accuracy (or inaccuracy) of a single block measurement, given the available data. In 50 random selections, the correct ranking was achieved in 68–74% of cases with the two-tier ranking. From a biological point of view, the four tumours examined in this study should be divided into two categories, rather than four. However, more than two categories might be expected in a study involving a large series of tumours; therefore, the frequency of obtaining the correct ranking in such a study would be closer to the 6–16% of cases that we obtained with the four-tier ranking. It is also of interest that the highest MVD value was not necessarily found in the periphery of the tumour, so that even if it were possible to select blocks from this region, this would not guarantee finding the highest MVD of the tumour. These results support the view that discrepancies between results obtained in different laboratories may be due to tumour heterogeneity.

When assessing the value of vascularity as a tumour prognostic indicator, the problem presented by tumour heterogeneity will remain whenever the tumour is large enough to be preserved in multiple blocks, as is often the case with lung carcinomas. The value of MVD as an index of angiogenesis and as a prognostic factor may depend, therefore, on the type and size of the tumours examined. It may be useful for carcinomas in-situ or for tumours that are relatively small, so that a single block will be representative of the tumour and sections will include the excision margins (Pazouki et al, 1997). For larger tumours, vascularity measured by MVD using pan-endothelial antibodies does not appear to be a reliable index of angiogenesis and is not likely to be useful as a routine assay using current methods.

Nevertheless, we found that intertumour variation in MVD values was statistically significant in spite of significant intratumour variation. Heterogeneity of any tumour parameter must be greater between tumours than within tumours for that parameter to be of clinical value. As this is the case for MVD, it is possible that this parameter may become informative when combined with other indices of angiogenesis and/or other methods to measure vascularity.

### ACKNOWLEDGEMENTS

We thank the Roy Castle Lung Foundation and the Cancer Research Campaign for financial support; Dr MW Myskow, Department of Histopathology, the Cardiothoracic Centre and Broadgreen Hospital, Liverpool, for providing the lung tissues; Dr M Bromley, Department of Histology, Paterson Institute for Cancer Research, Manchester, and Mr G Carmichael, Oral Medicine and Surgery Unit, Dundee University, for technical assistance.

### REFERENCES

Awwad HK, Naggar M, Mocktar N and Barsoum M (1986) Intercapillary distance measurement as an indicator of hypoxia in carcinoma of the cervix uteri. *Int J Radiat Oncol Biol Phys* **12**: 1329–1333

Axelsson K, Ljung B-ME, Moore DH, Thor AD, Chew KL, Edgerton SM, Smith HS and Mayall BH (1995) Tumour angiogenesis as a prognostic assay for invasive ductal breast carcinoma. *J Natl Cancer Inst* **87**: 997–1008

Bochner BH, Cote RJ, Weidner N, Groshen S, Chen SC, Skinner DG and Nichols PW (1995) Angiogenesis in bladder cancer: relationship between microvessel density and tumour prognosis. *J Natl Cancer Inst* **87**: 1603–1612

Carnochan P, Briggs JC, Westbury G and Davies AJ (1991) The vascularity of cutaneous melanoma: a quantitative histological study of lesions 0.85–1.25 mm in thickness. *Br J Cancer* **64**: 102–107

Chandrachud LM, Pendleton N, Chisholm DM, Horan MA and Schor AM (1997). Relationship between vascularity, age and survival in non-small cell lung cancer. *Br J Cancer* **76**: 1367–1375

De Jong JS, Vandiest PJ and Baak JPA (1995) Methods in laboratory investigation – heterogeneity and reproducibility of microvessel counts in breast cancer. *Lab Invest* **73**: 922–926

Delides GS, Venizelos J and Revesz L (1988) Vascularisation and curability of stage III and IV nasopharyngeal tumours. *J Cancer Res Clin Oncol* **114**: 321–332

Folkman J (1990) What is the evidence that tumours are angiogenesis dependent? *J Natl Cancer Inst* **82**: 4–6

Folkman J (1994) Angiogenesis and breast cancer. *J Clin Oncol* **12**: 441–443

Fox SB, Leek RD, Weekes MP, Whitehouse RM, Gatter KC and Harris AL (1995) Quantitation and prognostic value of breast cancer angiogenesis: comparison of microvessel density, Chalkley count, and computer image analysis. *J Pathol* **177**: 275–283

© Cancer Research Campaign 1998

Fisseler-Eckhoff A, Rothstein D and Muller KM (1996) Neovascularization in hyperplastic, metaplastic and potentially preneoplastic lesions of the bronchial mucosa. *Virchows Arch* **429**: 95–100

Gasparini G and Harris AL (1995) Clinical importance of the determination of tumour angiogenesis in breast carcinoma: much more than a new prognostic tool. *J Clin Oncol* **13**: 765–782

Hall NR, Fish DE, Hunt N, Goldin RD, Guillou PJ and Monson JRT (1992) Is the relationship between angiogenesis and metastasis in breast cancer real? *Surg Oncol* **1**: 223–229

Horak ER, Leek R, Klenk N, Le Jeune S, Smith K, Stuart N, Greenall M, Stepniewska K and Harris AL (1992) Angiogenesis, assessed by platelet/endothelial cell adhesion molecule antibodies, as indicator of node metastases and survival in breast cancer. *Lancet* **340**: 1120–1124

Kainz C, Speiser P, Wanner C, Obermair A, Tempfer C, Sliutz G, Reinthaller A and Breitenecker G (1995) Prognostic value of tumour microvessel density in cancer of the uterine cervix stage IB to IIB. *Anticancer Res* **15**: 1549–1551

Kolin A (1995) Tumour angiogenesis in human lung adenocarcinoma (correspondence). *Cancer* **76**: 151

Leedy DA, Trune DR, Kronz JD, Weidner N and Cohen JI (1994) Tumour angiogenesis, the p53 antigen, and cervical metastasis in squamous carcinoma of the tongue. *Otolaryngol Head Neck Surg* **111**: 417–422

Macchiarini P, Fontanini G, Hardin MJ, Squartini F and Angeletti CA (1992) Relation of neovascularisation to metastasis of non-small-cell lung cancer. *Lancet* **340**: 145–146

Mattern J, Koomagi R and Volm M (1995) Vascular endothelial growth factor and angiogenesis in non small lung cancer. *Int J Oncol* **6**: 1059–1062

Morphopoulus G, Pearson M, Ryder DJ, Howell A and Harris M (1996) Tumour angiogenesis as a prognostic marker in infiltrating lobular carcinoma of the breast. *J Pathol* **180**: 44–49

Pazouki S, Chisholm DM, Adi MM, Carmichael G, Farquharson M, Ogden GR, Schor SL and Schor AM (1997) The association between tumour progression and vascularity in the oral mucosa. *J Pathol* **183**: 39–43

Revesz L, Siracka E, Siracky J, Delides G and Pavlaki K (1989) Variation of vascular density within and between tumours of the uterine cervix and its predictive value for radiotherapy. *Int J Radiat Oncol Biol Phys* **16**: 1161–1163

Tahan SR and Stein AL (1995) Angiogenesis in invasive squamous cell carcinoma of the lip: tumour vascularity is not an indicator of metastatic risk. *J Cutan Pathol* **22**: 236–240

Van Hoef MEHM, Knox WF, Dhesi SS, Howell A and Schor AM (1993) Assessment of tumour vascularity as a prognostic factor in lymph node negative invasive breast cancer. *Eur J Cancer* **29A**: 1141–1145

Weidner N, Semple JP, Welch WR and Folkman J (1991) Tumour angiogenesis and metastasis – correlation in invasive breast carcinoma. *N Engl J Med* **324**: 1–8

Williams JK, Carlson GW, Cohen C, Derose PB, Hunter S and Jurkiewicz MJ (1994) Tumour angiogenesis as a prognostic factor in oral cavity tumours. *Am J Surg* **168**: 373–380

Zatterstrom UK, Brun E, Willen R, Kjellen E and Wennerberg J (1995) Tumour angiogenesis and prognosis in squamous cell carcinoma of the head and neck. *Head & Neck* **17**: 312–318

© Cancer Research Campaign 1998